# Response to Zhang and Lu

**DOI:** 10.1093/jnci/djag172

**Published:** 2026-05-28

**Authors:** Jun Okui, Kengo Nagashima, Satoru Matsuda

**Affiliations:** Department of Surgery, Keio University School of Medicine, Tokyo, Japan; Department of Biostatistics, Keio University School of Medicine, Tokyo, Japan; Biostatistics Unit, Clinical and Translational Research Center, Keio University Hospital, Tokyo, Japan; Department of Surgery, Keio University School of Medicine, Tokyo, Japan

We thank Drs. Zhang and Lu for their thoughtful comments regarding our study on evaluating conditional recurrence-free survival (RFS) after curative esophagectomy.^1^ We appreciate the opportunity to further clarify the interpretation and clinical implications of our findings.

First, we acknowledge that conditional survival analyses are inherently influenced by survivor bias. As the correspondents correctly point out, patients with advanced pathological disease who remain recurrence-free for several years likely represent a biologically favorable subgroup.^2^ However, our intention was neither to claim that recurrence risk becomes biologically identical across stages nor to advocate uniformly reducing surveillance intensity solely based on conditional RFS estimates. Rather, our findings demonstrate that prognostic discrimination based on baseline pathological stage dynamically attenuates over time. This phenomenon was consistently observed not only in conditional Kaplan–Meier estimates but also in the time-varying analyses using Aalen’s additive hazards model. Accordingly, we believe our results primarily provide a framework for dynamic risk reassessment during survivorship, rather than definitive recommendations for surveillance de-escalation.

Second, we also acknowledge the important issue of competing risks. In the present study, RFS was selected because it represents the most consistently available endpoint across the integrated randomized trial datasets.^3^ As correctly pointed out, noncancer mortality may increasingly contribute to late events during long-term follow-up. However, in the present study, analyses restricted to patients who experienced recurrence additionally demonstrated that the cumulative incidence of recurrence substantially plateaued after early postoperative years, particularly among patients with initially advanced-stage disease who remained recurrence-free. Therefore, we believe that the temporal convergence of hazards in conditional RFS likely reflects not only competing mortality but also genuine temporal changes in recurrence hazard itself. Nevertheless, we agree that future studies incorporating cumulative incidence function analyses separating recurrence and noncancer death would further refine survivorship risk stratification.^4^

Finally, we agree that the evolving incorporation of immune checkpoint inhibitors (ICIs) represents an important limitation of the present study.^5^ To preserve internal validity, we intentionally restricted the analysis to trials conducted largely within the chemotherapy and chemoradiotherapy era. As discussed in the article, this decision was made because phase 3 perioperative ICI trials remained limited and heterogeneous at the time of study planning. We agree that ICIs may alter recurrence dynamics and potentially increase the importance of late recurrence surveillance. Therefore, our findings should not be interpreted as directly generalizable to contemporary perioperative immunotherapy-treated populations. Rather, we view the present study as establishing a historical benchmark against which future ICI-era conditional survival analyses can be compared.

We sincerely appreciate the correspondents’ insightful comments, which have helped us contextualize both the strengths and limitations of our study, and highlight important directions for future research.

## Data Availability

No new data were generated or analyzed for this correspondence.
